# The influence of sentinel lymph node tumour burden on additional lymph node involvement and disease-free survival in cutaneous melanoma – a retrospective analysis of 392 cases

**DOI:** 10.1038/sj.bjc.6604407

**Published:** 2008-05-27

**Authors:** M Guggenheim, R Dummer, F J Jung, D Mihic-Probst, H Steinert, V Rousson, L E French, P Giovanoli

**Affiliations:** 1 Division of Plastic and Reconstructive Surgery, Department of Surgery, University Hospital Zurich, Zurich, Switzerland; 2 Department of Dermatology, University Hospital Zurich, Zurich, Switzerland; 3 Department of Pathology, Institute of Surgical Pathology, University Hospital Zurich, Zurich, Switzerland; 4 Division of Nuclear Medicine, Department of Medical Radiology, University Hospital Zurich, Zurich, Switzerland; 5 Biostatistics Unit, Institute for Social and Preventive Medicine, University of Lausanne, Lausanne, Switzerland

**Keywords:** melanoma, sentinel lymph node, recurrence, tumour load

## Abstract

Twenty per cent of sentinel lymph node (SLN)-positive melanoma patients have positive non-SLN lymph nodes in completion lymph node dissection (CLND). We investigated SLN tumour load, non-sentinel positivity and disease-free survival (DFS) to assess whether certain patients could be spared CLND. Sentinel lymph node biopsy was performed on 392 patients between 1999 and 2005. Median observation period was 38.8 months. Sentinel lymph node tumour load did not predict non-SLN positivity: 30.8% of patients with SLN macrometastases (⩾2 mm) and 16.4% with micrometastases (⩽2 mm) had non-SLN positivity (*P*=0.09). Tumour recurrences after positive SLNs were more than twice as frequent for SLN macrometastases (51.3%) than for micrometastases (24.6%) (*P*=0.005). For patients with SLN micrometastases, the DFS analysis was worse (*P*=0.003) when comparing those with positive non-SLNs (60% recurrences) to those without (17.6% recurrences). This difference did not translate into significant differences in DFS: patients with SLN micrometastasis, either with (*P*=0.022) or without additional positive non-SLNs (*P*<0.0001), fared worse than patients with tumour-free SLNs. The 2-mm cutoff for SLN tumour load accurately predicts differences in DFS. Non-SLN positivity in CLND, however, cannot be predicted. Therefore, contrary to other studies, no recommendations concerning discontinuation of CLND based on SLN tumour load can be deduced.

In the past decades, the incidence of cutaneous malignant melanoma has risen steadily, accompanied by an increase in mortality in male patients (Mackie *et al*, 2007). Due to major prevention efforts, the numbers seem to stabilise in younger age groups (Mackie *et al*, 2007). Early diagnosis has increased the proportion of thin melanomas with a greater likelihood for cure (McMasters and Swetter, 2003). However, the overall melanoma-specific survival remains unaffected despite all endeavours towards improving medical care. Much attention has been focused on the management of the regional lymph nodes (RLN) in melanoma patients. In this context, the surgical management strategies of the RLN have undergone considerable change in the past; with lymphatic mapping and sentinel lymph node (SLN) identification being the most relevant contribution (Cabanas, 1977; Wong *et al*, 1991; Morton *et al*, 1992). On the basis of the concept that the regional lymphatics serve as a barrier, temporarily trapping the orderly tumour spread from the primary site to more distant locations, it was proposed that the histopathological status of the SLN would accurately predict melanoma metastases (Reintgen *et al*, 1994; Thompson *et al*, 1995). Today, SNB is the most important staging tool, because the status of the SLN represents the most important prognostic factor for recurrence and survival for melanoma patients and identifies patients who might benefit from further therapy, such as completion lymph node dissection (CLND) and adjuvant interferon therapy (Morton *et al*, 1999; Balch *et al*, 2001; Hafner *et al*, 2004; Eggermont *et al*, 2007).

Nevertheless, the impact of sentinel lymph node biopsy (SLNB) on survival remains unclear. Recently, Morton *et al* (2006) reported an increased disease-free survival (DFS) with no significant impact on over-all survival, raising the question whether lymph node dissection is necessary in case of a positive SLN. The identification of predictive factors for non-SLN positivity is a challenge to spare SLN-positive patients the morbidity of CLND. Unlike the situation for cutaneous melanoma, widely accepted guidelines exist for breast cancer, which no longer recommend CLND in patients with an SLN submicrometastasis (<0.2 mm), as they are highly unlikely to recur regionally (Fournier *et al*, 2004; Rutgers, 2004). We communicate our SLNB experience during a 7-year period, particularly focusing on SLN tumour load, non-SLN positivity and patterns of tumour recurrence.

## PATIENTS AND METHODS

### Patients

In all, 392 patients with cutaneous malignant melanoma underwent SLNB, from the introduction of the method in our institution from October 1999 to December 2005, and were followed up until 31 September 2006. The median period of observation was 38.8 months.

### Technique of SLN identification, wide excision and sentinel lymph node biopsy

Most patients had already undergone diagnostic excision of the tumour. All patients underwent WE wide excision of the primary tumour with a safety margin of 1 cm for Breslow thickness below 2 mm and a safety margin of 2 cm for Breslow thickness above 2 mm, in accordance with Swiss guidelines (Dummer *et al*, 2005). Neither the triple technique used to identify and remove the SLNs nor the methodology employed for pathological analysis of the removed lymph nodes differs from that previously described in the literature (Hafner *et al*, 2004; Morton *et al*, 2005). Consistent with published guidelines (Cochran *et al*, 2000), SLNB was recommended for pathological staging of the RLN in patients with a minimal Breslow of 1.00 mm and no clinical or radiological evidence of melanoma metastasis at the time of diagnosis. SLNB was equally performed in 15 patients with a smaller tumour thickness, for whom the referring dermatologist urged staging, either because the Breslow value was only slightly below 1.00 mm or histological review revealed aggravating factors. Completion lymph node dissection was recommended for positive SLNs, according to the Augsburg Consensus guidelines (Cochran *et al*, 2000). Clinicopathologic characteristics were analysed, including sex, age, location of the primary tumour, Breslow value and size of metastatic depots. Disease-free survival and primary recurrences were determined separately for SLN-positive and SLN-negative patients according to the anatomic location of the first recurrence. Local recurrences were defined as satellite recurrence within or up to 3 cm around the wide excision scar; in-transit recurrence was defined as recurrence in the dermal lymphatics between the site of the excised primary tumour and the regional nodal basin (RNB). Recurrences within the staged regional nodal basin were considered RNB recurrences and distant recurrences as distant skin, nodal or systemic recurrences beyond the staged RNB. Seven patients with positive SLNB refused to undergo CLND. They were counted as SLN-positive and were continued to be followed up at the Department of Dermatology, but their data were not included with the CLND group for further analysis.

### Follow-up

Patients were all followed up in our outpatient clinic, until aftercare for all surgical procedures, including the complications thereof, could safely be terminated. Oncological follow-up was performed in the Department of Dermatology according to Swiss national guidelines using a standardised sequence of imaging techniques (Dummer *et al*, 2005). Recurrences were registered and patients treated according to the site of recurrence, surgically, systemically or by radiotherapy.

### Technique of histopathological SLN workup

After 1 day of fixation, the SLN was bisected along the long axis of the hilar region. If the SLN was thick, the two halves were further cut into 2-mm thick sections. Depending on its size, the bisected node was embedded in one or more paraffin blocks. Paraplast sections at five intervals of 50 *μ*m were prepared from each paraffin block. From each paraplast section, four slides were made and stained with haematoxylin–eosin and immune stained with antibodies to S-100, HMB-45 and Pan Melanoma Plus acoording the EORTC recommendations for working up melanoma SLNs (Cook *et al*, 2003). Haematoxylin–eosin- and immune-stained sections of all samples were reviewed by one experienced pathologist (DM). There were four different diagnoses based on the recommendations of the International Union against Cancer: (i) no tumour, (ii) isolated tumour cells, (iii) micrometastasis (<2 mm) and (iv) macrometastasis (>2 mm) (Hermanek *et al*, 1999). As no significant differences between patients with isolated tumour cells and patients with micrometastasis were found, we have summarised both groups under the heading of micrometastasis for this analysis, as described in previous studies (Carlson *et al*, 2003; Pearlman *et al*, 2006; Roka *et al*, 2008).

### Statistical analysis

Statistical analysis was performed using SPSS 13. Statistical comparison between two groups of patients was done using a *t*-test for continuous variables and a *χ*^2^ test for categorical variables. Breslow thickness was log-transformed to reach an approximate normal distribution. Comparison of groups with respect to the end point ‘time to recurrence’ has been done using Kaplan–Meier curves and a log-rank test. For the relationship between a continuous variable (like log Breslow thickness) and time to recurrence, a Cox regression has been calculated. *P*-values below 0.05 were considered as significant.

## RESULTS

### Patient characteristics and regional lymph node basin status

Baseline patient characteristics are summarised in Table 1. Male patients were significantly older at the time of diagnosis (*P*<0.0001, *t*-test). Breslow values for both sexes do not differ significantly (*P*=0.60, *t*-test). Analysis of the location of the primary tumour correlated well with gender-specific differences previously described (Cabanas, 1977), with the lower extremity as the most common melanoma site in females and the trunk in males (*P*<0.0001, *χ*^2^ test). A total of 470 hot nodes were identified; on average 1.2 SLN (range 1–3) were removed per patient. A total of 427 RNBs were staged in our 392 patients; as in 31 patients, lymphocintigraphy identified SLNs in two RNBs, and two patients had hot nodes in three RNBs simultaneously. We staged 221 axillary RNBs, 146 in the groin and 52 in the head and neck area. Intercalated nodes (popliteal fossa, cubital fossa, medial bicipital sulcus, lateral chest wall) were identified in eight patients (2%); in six (1.5%) of them in conjunction with either additional inguinal or axillary SLNs. All of these intercalated nodes were tumour-free. The rate of major complications mandating either additional surgery or stationary hospital care was 2.3%. A total of 114 positive SLNs (24.5%) in 107 patients (27.3%) were found. Micrometastatic tumour deposits were found in 66 of 107 (61.7%) patients with positive SLNs, whereas macrometastases were found in 41 (38.3%) patients. Out of 66 patients with micrometastases, 11 presented isolated tumour cells (10.3%). Table 2 displays a comparison, stratified by age, sex and Breslow thickness of SLN-positive and SLN-negative patients. Neither gender was significantly associated with a higher rate of positive SLNs nor, consequently, with a gender-dependant significantly worse prognosis (*P*=0.84, *χ*^2^ test). No statistically significant differences were evident for median and mean age in SLN-positive and SLN-negative groups (*P*=0.23, *t*-test). Both mean and median Breslow thickness were significantly greater in the SLN-positive group (*P*<0.0001, *t*-test). Stratification of the SLN-positive patients according to Breslow values (Table 3) revealed increasing rates of SLN positivity proportional to greater thickness of the primary tumour. Influence of the location of the primary tumour on SLN positivity was statistically insignificant (Table 4) (*P*=0.38, *χ*^2^ test). Completion lymph node dissection was recommended for all 107 patients with positive SLNs. In all, 100 (93.5%) underwent the procedure, seven (6.5%) refused and their data were not included for the outcome analysis of the SLN-positive group.

### SLN-positive patients – CLND results

We performed a total of 102 CLNDs on 100 patients, as one patient had bilateral axillary CLNDs and one patient had bilateral groin CLNDs simultaneously. In all, 46.1% of all CLNDs were axillary (47/102), 42.2% were in the groin (43/102) and 11.8% were neck dissections (12/102). No additional positive non-SLN was found in 78% (78/100) of the CLNDs, whereas 22% (22/100) had additional positive nodes. Sentinel lymph node tumour load did not effectively predict non-SLN positivity in CLND; 30.8% (12/39) of the CLND patients with a macrometastasis in their sentinel had further non-SLN positivity. In comparison, 16.4% (10/61) of those patients with an SLN micrometastasis had a positive CLND (*P*=0.09, *χ*^2^ test).

### SLN-positive patients – melanoma recurrence

**Figure 1:**
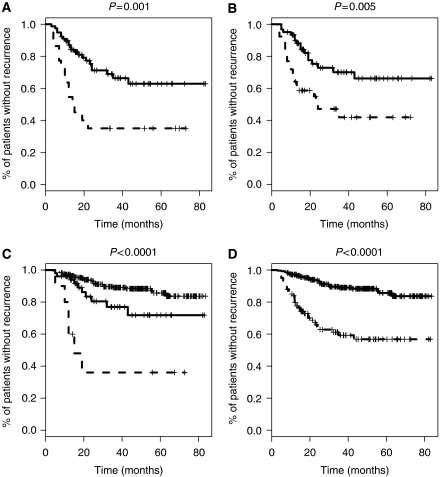
Comparison of Kaplan–Meier curves for disease-free survival. (**A**) Sentinel lymph node-positive patients without (solid line) and with (dashed line) additional positive non-SLNs, (**B**) SLN-positive patients with SLN micrometastases (solid line) and SLN macrometastases (dashed line), (**C**) SLN-negative patients (solid line), SLN-positive patients with micrometastases without (bold solid line) and with additional positive non-SLNs in CLND (dashed line), and (**D**) SLN-negative (solid line) and SLN-positive (dashed line) patients. *P*-values were calculated using a log-rank test.

Sixty-five per cent (65/100) of patients, who underwent CLND, remained disease-free; 35% (35/100) presented a tumour relapse: median time to recurrence was 12.5 months (range 3–43 months). Primary sites of recurrence were local or in-transit (9/35, 25.7%), nodal (11/35, 31.4%) and distant (15/35, 42.9%). Patients without further positive non-SLNs had significantly less recurrent disease (26.9%, 21/78) compared to those with additional positive non-SLNs in CLND (14/22, 63.3%) (*P*=0.001, log-rank test) (cf. Table 5 and Figure 1A). The effect of tumour load on recurrence was pronounced; in general, recurrence rates in the SLN-positive and SLN-negative groups were 35.0 and 10.9% respectively, the difference being highly significant (*P*<0.0001, log-rank test) (cf. Figure 1D). Among the SLN-positive patients, tumour recurrences after a positive SLNB were more than twice as frequent for SLN macrometastases (51.3%, 20/39) than for micrometastases (24.6%, 15/61), the difference being significant in a DFS analysis (*P*=0.005, log-rank test) (cf. Figure 1B). Stratified by the size of SLN metastasis and the number of additional positive non-SLNs in the CLND, the impact of SLN tumour load becomes even more evident: 51 of the 78 patients with no further positive lymph node in the CLND had micrometastatic tumour depots in their SLNs; nine of them (17.6%) suffered a relapse. This rate was significantly higher and more than doubled for the 27 patients in whom the SLN, although remaining the only positive lymph node even after CLND, harboured a macrometastasis: 12 patients (44.4%) exhibited tumour recurrences (*P*=0.009, log-rank test). Even more impressive was the correlation between additional positive non-SLNs in CLND and tumour recurrence: 66.7% (8/12) of all patients with SLN macrometastases and 60% (6/10) of patients with SLN micrometastases who had positive non-SLNs in CLND relapsed during the mean follow-up period of 38.8 months. The influence of additional positive non-SLNs aggravated tumour recurrence only by increasing total tumour burden and did not exert more prognostic influence than SLN tumour burden alone: patients with SLN macrometastases and positive non-SLNs did not have significantly more tumour recurrences compared to those with SLN micrometastases and positive non-SLNs (*P*=0.60). Sentinel lymph node tumour burden did, however, influence recurrence significantly when analysing micro- and macrometastases separately: although for patients with SLN macrometastases, the difference in the development of tumour recurrences did not differ significantly (*P*=0.17) between patients with (recurrence 66.7%, 8/12) or without (recurrence 44.4%, 12/27) additional non-SLNs in CLND, for patients with SLN micrometastases, the DFS analysis was significantly worse when comparing those with additional non-SLNs (60% recurrences, 6/10) to those without (17.6% recurrences, 9/51) (*P*=0.003, log-rank test). This difference did not, however, correspond to significant differences in DFS: both patients with SLN micrometastasis either with (*P*=0.022, log-rank test) or without additional positive non-SLNs (*P*<0.0001, log-rank test) fared significantly worse than patients with tumour-free SLNs (cf. Figure 1C).

For Breslow thickness, another indicator for tumour load, there was also correlation with tumour recurrence; for intermediate thickness melanomas (Breslow 1–4 mm), the rate of recurrence after positive SLNB remained within similar margins (Table 3). In 27.5% (11/40) of the patients with a primary tumour thickness of 1–2 mm, the tumour relapsed, as well as in 33.3% (11/33) of the patients with a primary Breslow of 2–4 mm. The rate of recurrence, however, increased sharply for positive SLNs associated with a primary melanoma thicker than 4 mm; every second (50%, 12/24) recurred after CLND. The percentage of micro- and macrometastases, however, remained fairly constant and did not increase proportionally with Breslow thickness (Table 3) (*P*=0.23, *t*-test).

### Negative patients

During the median period of observation of 38.8 months, 89.1% (254) of the 285 negative patients showed DFS. In all, 10.9% (Van Akkooi *et al*, 2006), however, exhibited recurrences, and the SLNB was therefore considered false-negative (FN). Identical FN rates were noted for distant (4.2%, 12/31) and nodal primary recurrence. Local or in-transit relapse was seen in 2.5% (7/31). All of the RNBs, in which SLNB was performed, yielded similar rates of FN results. In all, 7.7% (17/221) of all staged axillary RNBs produced FN results, 6.2% (9/146) were from SLNBs in the groin and 9.6% (5/52) came from head and neck RNBs. Median time to recurrence for FN SLNB patients was 23 months; 24 month for primary distant relapse, 19 for primary nodal failure and 16 for local and in-transit recurrence.

## DISCUSSION

In this single centre retrospective analysis, we reviewed our experience with SLNB in cutaneous melanoma. Our well-characterised patient population was treated and followed using a structured algorithm and compared well with other series published. Our main objective was to study the correlation between SLN tumour load and further non-SLN positivity as well as DFS.

Consistent with our own data (78%), 67–90% of SLN-positive patients do not have further non-SLNs that contain tumour deposits in the CLND specimens (Reintgen *et al*, 1994; Carlson *et al*, 2003; Van Akkooi *et al*, 2006). As a consequence, the majority of SLN-positive patients undergo unnecessary surgery associated with considerable morbidity (Guggenheim *et al*, 2008). Therefore, several authors have tried to identify patient, tumour and SLN characteristics predicting further non-SLN positivity to safely avoid CLND (Carlson *et al*, 2003; Van Akkooi *et al*, 2006; Scolyer *et al*, 2007). Although CLND has not yet been proven to positively influence overall melanoma-specific survival, Cascinelli *et al* (2006) have recently shown that CLND is necessary to achieve the best assessment of prognosis of stage IB and II melanoma and to identify those patients who, having only positive sentinel nodes and negative non-sentinel nodes, have a good prognosis. Although previous studies have failed to consistently identify the same clinicopathological features as indicators for additional non-SLN positivity upon CLND or for DFS (Scolyer *et al*, 2007), SLN tumour load, nevertheless, was uniformly confirmed by all of these studies as prognosticator for non-SLN positivity and recurrence. Thus, we focus our analysis on this characteristic.

There is considerable debate as to how to stratify SLN tumour burden; Satzger *et al* (2007) found that isolated immunohistochemically positive tumour cells are without prognostic significance and DFS of these patients did not differ from that of SLN-negative patients, an observation that is supported in a broader sense by Van Akkooi *et al* (2006). In their study, no patient with an SLN tumour load of <0.1 mm had additional non-SLN positivity upon CLND, and 5-year overall survival was 100%. On the basis of these data, they suggested that such patients may be considered SLN-negative and should be spared CLND. A similar observation, albeit with a cutoff <0.2 mm, was made by Govindarajan *et al* (2007). Both studies did, however, either not reach statistical significance (Van Akkooi *et al*, 2006) or the study population was relatively small (Govindarajan *et al*, 2007). Yet another cutoff based on a novel micromorphometric classification, albeit this time at 1 mm above submicroscopic levels, was proposed by Starz *et al* (2004). In his studies, patients with deposits <1 mm had survival rates not significantly different from those of patients with tumour-free SLNs. As these results proved to be difficult to reproduce, however, all these observations are contested by other authors (Scheri *et al*, 2007; Scolyer *et al*, 2007). Scheri *et al* (2007) found that 12% of their patients with isolated tumour cells had further positive non-SLNs in their CLND specimens and that their melanoma-specific survival was significantly worse than in those patients with negative SLNs.

The failure to predict the necessicity of CLND based on submicroscopic SLN tumour load is demonstrated by several studies; Carlson *et al* (2003) reported that 22.6% of patients with isolated tumour cells had further positive non-SLNs upon CLND. Although the numbers are too small to reach significance, our own data from patients with isolated tumour cells indicate that indeed submicroscopic cutoffs and micromorphometric classifications may not contribute much towards clarifying behavioral and prognostic differences according to SLN tumour burden. Of the 11 patients with isolated tumour cells in our series, only one (9.1%) had additional positive non-SLNs, but three (27.3%) had tumour recurrence during follow-up. The cutoff separating micrometastases from macrometastases at 2 mm, as put forth by Hermanek *et al* (1999), however, may allow more promising conclusions. Several authors have used this cutoff in analysing their study populations. Despite the fact that 6% of the patients with micrometastases (isolated tumour cells not differentiated) in their SLNs had a positive CLND, Pearlman *et al* (2006) found that their 5-year survival was at 85% essentially the same as that of patients with a negative SLNB. Carlson *et al* (2003) have made a similar observation: even though SLN tumour burden was not predictive of non-SLN positivity, the 3-year overall survival for patients with SLN tumour burden ⩽2 mm (including isolated tumour cells) was significantly higher than that for those with SLN tumour deposits of >2 mm (90 *vs* 57%), irrespective of whether patients had positive CLNDs or not. Roka *et al* (2008) were able to partly confirm this: even though no significant association between SLN tumour load and non-SLN positivity was found, the rate of DFS for patients with an SLN tumour burden of >2 mm was significantly worse. Similar observations come from a study by Ranieri *et al* (2002), albeit with a cutoff at 3 mm. Our own data confirm these results in part: SLN tumour burden with a cutoff at 2 mm was indeed a significant prognosticator for tumour recurrence (*P*=0.005, log-rank test), with the rates of relapse during the median observation period more than twice as frequent for SLN macrometastases (51.3%) as for micrometastases (24.6%). Moreover, even though there was no association between SLN tumour burden and additional non-SLN positivity, there was a clear statistical trend (*P*=0.09) in our study indicating that patients with higher SLN tumour burden might be associated twice as likely with non-SLN positivity. This finding confirms a similar trend demonstrated by Roka *et al* (2008) that may reach statistical significance once analysed in larger study populations. The rates for positive CLNDs were not significantly different for SLN macrometastases and micrometastases. This is in accordance with other studies in which reproducible prediction of non-SLN positivity on the basis of SLN tumour burden remained elusive (Ranieri *et al*, 2002; Carlson *et al*, 2003; Pearlman *et al*, 2006; Roka *et al*, 2008). Additional positive non-SLNs upon CLND are widely recognised to adversely influence prognosis (Carlson *et al*, 2003). In our study, tumour recurrences were significantly more frequent in patients with additional positive non-SLNs in CLND than in those who did not have a positive CLND. Although our study confirms that predicting non-SLN positivity on the basis of SLN tumour load is unreliable, it demonstrates that SLN tumour burden has an impact on DFS. Recent experimental studies using melanoma cell lines in mice have impressively shown that melanoma cells can switch their transcriptional profile from an invasive migrating one to a proliferative profile associated with melanocytic differentiation (Hoek *et al*, 2008). We hypothesise that the evaluation of the invasive markers in melanoma metastases might improve the predictive accuracy of the SN status.

Today, neither clinicopathological nor histomorphometrical characteristics reliably and reproducibly predict non-SLN positivity in CLND. However, in accordance with several other authors, our study supports the observation that the cutoff at 2 mm for SLN tumour load serves to accurately predict differences in DFS. In contrast to other studies, however, patients with SLN tumour burden ⩽2 mm did have DFS significantly worse than those with tumour-free SLNs. Although far from allowing conclusions, our study illustrates that we do not as of yet sufficiently understand what constitutes relevant nodal disease. Even though CLND has not been proven to improve survival, pending the results of MSLT-II, no clinical recommendations concerning the discontinuation of CLND based on SLN tumour load can be deduced.

## Figures and Tables

**Table 1:** Characteristics of 392 patients undergoing SLNB at university hospital Zurich during 1999–2005

***n*=392, sex**	**Age (years)**	**Breslow (mm)**	**Primary tumour location**
168, female (42.9%)	48.7 (mean)	2.45 (mean)	Head/neck, 20 (11.9%)
	49 (median)	1.85 (median)	Upper extremity, 41 (24.4%)
	16–79 (range)	0.4–17 (range)	Trunk, 25 (14.9%)
		Unknown *n*=4	Lower extremity, 82 (48.8%)
			
224, male (57.1%)	57.1 (mean)	2.54 (mean)	Head/neck, 31 (13.8%)
	61 (median)	1.90 (median)	Upper extremity, 60 (26.8%)
	18–85 range	0.4–14 (range)	Trunk, 95 (42.4%)
		Unknown *n*=6	Lower extremity, 36 (16.1%)
			Unknown, 2 (0.9%)

SLN=sentinel lymph node.

**Table 2:** Characteristics of sentinel-positive *vs* sentinel-negative patients

**Patients=392**	**Age (years)**	**Breslow (mm)**	**Sex**
285 SLN-neg (72.7%)	53 (mean)	1.77 (mean)	Male, 56.8% (*n*=162)
	57 (median)	1.80 (median)	Female, 43.2% (*n*=123)
	16–85 (range)	(0.4–14)	
		(unknown *n*=9)	
			
107 SLN-pos (27.3%)	55 (mean)	3.39 (mean)	Male, 57.9% (*n*=62)
	58 (median)	2.21 (median)	Female, 42.1% (*n*=45)
	20–80 (range)	(0.82–17)	
		(unknown *n*=1)	

SLN=sentinel lymph node.

**Table 3:** SLN positivity, type of metastasis and recurrence stratified according to Breslow thickness of primary tumour, for patients subject to CLND

**Breslow (mm)**	**Patient (%) (*n*=385)**	**Positive patient (%)**	**Micrometastasis in SLN (as % of positive SLN)**	**Macrometastasis in SLN (as % of positive SLN)**	**Recurrence after CLND (%)**
Unknown	2.6 (*n*=10)	10 (*n*=1)	100 (*n*=1)	0	100 (*n*=1) L/i=1
<1	3.6 (*n*=14)	14.3 (*n*=2)	100 (*n*=2)	0	0
1–2	48.1 (*n*=185)	21.6 (*n*=40)	60 (*n*=24)	40 (*n*=16)	27.5 (*n*=11) L/i=2 * n*=6 * d*=3
2–4	31.7 (*n*=122)	27 (*n*=33)	66.7 (*n*=22)	33.3 (*n*=11)	30.3 (*n*=11) L/i=5 * n*=3 * d*=3
>4	14.0 (*n*=54)	44.4 (*n*=24)	54.2 (*n*=13)	45.8 (*n*=11)	50 (*n*=12) L/i=1 * n*=2 * d*=9

CLND=completion lymph node dissection; d=distant; L/I=local/intransit; n=nodal; SLN=sentinel lymph node.

The seven patients who refused CLND are excluded from the total number of patients.

**Table 4:** Sentinel positivity as a function of the location of the primary tumour

**Sex of positive patients**	**Location of primary tumour (%)**	**Location of primary tumour in SLN-pos patients**
Female (*n*=45)	Head/neck, 20 (11.9%)	Head/neck, 13.3% (*n*=6)
	Upper extremity, 41 (24.4%)	Upper extremity, 17.8% (*n*=8)
	Trunk, 25 (14.9%)	Trunk, 20% (*n*=9)
	Lower extremity, 82 (48.8%)	Lower extremity, 48.9% (*n*=22)
		
Male (*n*=62)	Head/neck, 31 (13.8%)	Head/neck, 11.3% (*n*=7)
	Upper extremity, 60 (26.8%)	Upper extremity, 24.2% (*n*=15)
	Trunk, 95 (42.4%)	Trunk, 37.1% (*n*=23)
	Lower extremity, 36 (16.1%)	Lower extremity, 27.4% (*n*=17)

SLN=sentinel lymph node.

**Table 5:** Number of positive non-SLNs in CLND and associated tumour recurrence

**No. of positive non-SLNs in CLND**	**% Patients (*n*=100)**	**% Recurrence (*n*=34)**
0	78 (*n*=78)	26.9 (*n*=21)
1	8 (*n*=8)	75.0 (*n*=6)
2	7 (*n*=7)	57.1 (*n*=4)
⩾3	7 (*n*=7)	57.1 (*n*=4)

CLND=completion lymph node dissection; SLN=sentinel lymph node.

## References

[bib1] Balch CM, Soong SJ, Gershenwald JE, Thompson JF, Reintgen DS, Cascinelli N, Urist M, Mcmasters KM, Ross MI, Kirkwood JM, Atkins MB, Thompson JA, Coit DG, Byrd D, Desmond R, Zhang Y, Liu PY, Lyman GH, Morabito A (2001) Prognostic factors analysis of 17,600 melanoma patients: validation of the American Joint Committee on Cancer melanoma staging system. J Clin Oncol 19: 3622 11504744 10.1200/JCO.2001.19.16.3622

[bib2] Cabanas RM (1977) An approach for the treatment of penile carcinoma. Cancer 39: 456 837331 10.1002/1097-0142(197702)39:2<456::aid-cncr2820390214>3.0.co;2-i

[bib3] Carlson GW, Murray DR, Lyles RH, Staley CA, Hestley A, Cohen C (2003) The amount of metastatic melanoma in a sentinel lymph node: does it have prognostic significance? Ann Surg Oncol 10: 575 12794026 10.1245/aso.2003.03.054

[bib4] Cascinelli N, Bombardieri E, Bufalino R, Camerini T, Carbone A, Clemente C, Lenisa L, Mascheroni L, Maurichi A, Pennacchioli E, Patuzzo R, Santinami M, Tragni G (2006) Sentinel and nonsentinel node status in stage IB and II melanoma patients: two-step prognostic indicators of survival. J Clin Oncol 24: 4464 16983115 10.1200/JCO.2006.06.3198

[bib5] Cochran AJ, Balda BR, Starz H, Bachter D, Krag DN, Cruse CW, Pijpers R, Morton DL (2000) The Augsburg Consensus. Techniques of lymphatic mapping, sentinel lymphadenectomy, and completion lymphadenectomy in cutaneous malignancies. Cancer 89: 236 10918150

[bib6] Cook MG, Green MA, Anderson B, Eggermont AM, Ruiter DJ, Spatz A, Kissin MW, Powell BW (2003) The development of optimal pathological assessment of sentinel lymph nodes for melanoma. J Pathol 200: 314 12845627 10.1002/path.1365

[bib7] Dummer R, Panizzon R, Bloch PH, Burg G (2005) Updated Swiss guidelines for the treatment and follow-up of cutaneous melanoma. Dermatology 210: 39 10.1159/000081482 15604544

[bib8] Eggermont AMSS, Santinami M, Kruit W, Testori A, Marsden J, Punt C, Hauschild A, Gore M, Keilholz U, EORTC Melanoma group (2007) EORTC 18991: Long-term adjuvant pegylated interferon-alpha2b (PEG-IFN) compared to observation in resected stage III melanoma, final results of a randomized phase III trial. J Clin Oncol; 2007 ASCO Annual Meeting Proceedings Part I 25No. 18S: 8517194908

[bib9] Fournier K, Schiller A, Perry RR, Laronga C (2004) Micrometastasis in the sentinel lymph node of breast cancer does not mandate completion axillary dissection. Ann Surg 239: 859 15166965 10.1097/01.sla.0000128302.05898.a7 PMC1356294

[bib10] Govindarajan A, Ghazarian DM, Mccready DR, Leong WL (2007) Histological features of melanoma sentinel lymph node metastases associated with status of the completion lymphadenectomy and rate of subsequent relapse. Ann Surg Oncol 14: 906 17136471 10.1245/s10434-006-9241-3

[bib11] Guggenheim MM, Hug U, Jung FJ, Rousson V, Aust MC, Calcagni M, Kunzi W, Giovanoli P (2008) Morbidity and recurrence after completion lymph node dissection following sentinel lymph node biopsy in cutaneous malignant melanoma. Ann Surg 247: 687 18362633 10.1097/SLA.0b013e318161312a

[bib12] Hafner J, Schmid MH, Kempf W, Burg G, Kunzi W, Meuli-Simmen C, Neff P, Meyer V, Mihic D, Garzoli E, Jungius KP, Seifert B, Dummer R, Steinert H (2004) Baseline staging in cutaneous malignant melanoma. Br J Dermatol 150: 677 15099363 10.1111/j.0007-0963.2004.05870.x

[bib13] Hermanek P, Hutter RV, Sobin LH, Wittekind C (1999) International union against cancer. classification of isolated tumor cells and micrometastasis. Cancer 86: 2668 10594862

[bib14] Hoek KS, Eichhoff OM, Schlegel NC, Dobbeling U, Kobert N, Schaerer L, Hemmi S, Dummer R (2008) *In vivo* switching of human melanoma cells between proliferative and invasive states. Cancer Res 68: 650 18245463 10.1158/0008-5472.CAN-07-2491

[bib15] Mackie RM, Bray C, Vestey J, Doherty V, Evans A, Thomson D, Nicolson M (2007) Melanoma incidence and mortality in Scotland 1979–2003. Br J Cancer 96: 1772 17533392 10.1038/sj.bjc.6603801 PMC2359933

[bib16] McMasters KM, Swetter SM (2003) Current management of melanoma: benefits of surgical staging and adjuvant therapy. J Surg Oncol 82: 209 12619066 10.1002/jso.10216

[bib17] Morton DL, Cochran AJ, Thompson JF, Elashoff R, Essner R, Glass EC, Mozzillo N, Nieweg OE, Roses DF, Hoekstra HJ, Karakousis CP, Reintgen DS, Coventry BJ, Wang HJ (2005) Sentinel node biopsy for early-stage melanoma: accuracy and morbidity in MSLT-I, an international multicenter trial. Ann Surg 242: 302 16135917 10.1097/01.sla.0000181092.50141.fa PMC1357739

[bib18] Morton DL, Thompson JF, Cochran AJ, Mozzillo N, Elashoff R, Essner R, Nieweg OE, Roses DF, Hoekstra HJ, Karakousis CP, Reintgen DS, Coventry BJ, Glass EC, Wang HJ (2006) Sentinel-node biopsy or nodal observation in melanoma. N Engl J Med 355: 1307 17005948 10.1056/NEJMoa060992

[bib19] Morton DL, Thompson JF, Essner R, Elashoff R, Stern SL, Nieweg OE, Roses DF, Karakousis CP, Mozzillo N, Reintgen D, Wang HJ, Glass EC, Cochran AJ (1999) Validation of the accuracy of intraoperative lymphatic mapping and sentinel lymphadenectomy for early-stage melanoma: a multicenter trial. Multicenter Selective Lymphadenectomy Trial Group. Ann Surg 230: 453 10522715 10.1097/00000658-199910000-00001 PMC1420894

[bib20] Morton DL, Wen DR, Wong JH, Economou JS, Cagle LA, Storm FK, Foshag LJ, Cochran AJ (1992) Technical details of intraoperative lymphatic mapping for early stage melanoma. Arch Surg 127: 392 1558490 10.1001/archsurg.1992.01420040034005

[bib21] Pearlman NW, McCarter MD, Frank M, Hurtubis C, Merkow RP, Franklin WA, Gonzalez R, Lewis K, Roaten JB, Robinson WA (2006) Size of sentinel node metastases predicts other nodal disease and survival in malignant melanoma. Am J Surg 192: 878 17161112 10.1016/j.amjsurg.2006.08.062

[bib22] Ranieri JM, Wagner JD, Azuaje R, Davidson D, Wenck S, Fyffe J, Coleman JJR (2002) Prognostic importance of lymph node tumor burden in melanoma patients staged by sentinel node biopsy. Ann Surg Oncol 9: 975 12464589 10.1007/BF02574515

[bib23] Reintgen D, Cruse CW, Wells K, Berman C, Fenske N, Glass F, Schroer K, Heller R, Ross M, Lyman G, Et A (1994) The orderly progression of melanoma nodal metastases. Ann Surg 220: 759 7986143 10.1097/00000658-199412000-00009 PMC1234478

[bib24] Roka F, Mastan P, Binder M, Okamoto I, Mittlboeck M, Horvat R, Pehamberger H, Diem E (2008) Prediction of non-sentinel node status and outcome in sentinel node-positive melanoma patients. Eur J Surg Oncol 34: 82 17360144 10.1016/j.ejso.2007.01.027

[bib25] Rutgers EJ (2004) Sentinel node micrometastasis in breast cancer. Br J Surg 91: 1241 15382100 10.1002/bjs.4800

[bib26] Satzger I, Volker B, Meier A, Schenck F, Kapp A, Gutzmer R (2007) Prognostic significance of isolated HMB45 or Melan A positive cells in Melanoma sentinel lymph nodes. Am J Surg Pathol 31: 1175 17667539 10.1097/PAS.0b013e3180341ebc

[bib27] Scheri RP, Essner R, Turner RR, Ye X, Morton DL (2007) Isolated tumor cells in the sentinel node affect long-term prognosis of patients with melanoma. Ann Surg Oncol 14: 2861 17882497 10.1245/s10434-007-9472-y

[bib28] Scolyer RA, Murali R, Gershenwald JE, Cochran AJ, Thompson JF (2007) Clinical relevance of melanoma micrometastases in sentinel nodes: too early to tell. Ann Oncol 18: 806 17389530 10.1093/annonc/mdm081

[bib29] Starz H, Siedlecki K, Balda BR (2004) Sentinel lymphonodectomy and s-classification: a successful strategy for better prediction and improvement of outcome of melanoma. Ann Surg Oncol 11: 162S 15023745 10.1007/BF02523622

[bib30] Thompson JF, Mccarthy WH, Bosch CM, O'Brien CJ, Quinn MJ, Paramaesvaran S, Crotty K, McCarthy SW, Uren RF, Howman-Giles R (1995) Sentinel lymph node status as an indicator of the presence of metastatic melanoma in regional lymph nodes. Melanoma Res 5: 255 7496161 10.1097/00008390-199508000-00008

[bib31] Van Akkooi AC, De Wilt JH, Verhoef C, Schmitz PI, Van Geel AN, Eggermont AM, Kliffen M (2006) Clinical relevance of melanoma micrometastases (<0.1 mm) in sentinel nodes: are these nodes to be considered negative? Ann Oncol 17: 1578 16968875 10.1093/annonc/mdl176

[bib32] Wong JH, Cagle LA, Morton DL (1991) Lymphatic drainage of skin to a sentinel lymph node in a feline model. Ann Surg 214: 637 1953118 10.1097/00000658-199111000-00015 PMC1358621

